# Gap Junction Channelopathies and Calmodulinopathies. Do Disease-Causing Calmodulin Mutants Affect Direct Cell–Cell Communication?

**DOI:** 10.3390/ijms22179169

**Published:** 2021-08-25

**Authors:** Camillo Peracchia

**Affiliations:** Department of Pharmacology and Physiology, School of Medicine and Dentistry, University of Rochester, Rochester, NY 14642-8711, USA; camillo.peracchia@gmail.com

**Keywords:** gap junctions, connexins, channel gating, calcium, calmodulin, cell communication, cell-to-cell channels, cell coupling, cell uncoupling, channelopathies, calmodulinopathies

## Abstract

The cloning of connexins cDNA opened the way to the field of gap junction channelopathies. Thus far, at least 35 genetic diseases, resulting from mutations of 11 different connexin genes, are known to cause numerous structural and functional defects in the central and peripheral nervous system as well as in the heart, skin, eyes, teeth, ears, bone, hair, nails and lymphatic system. While all of these diseases are due to connexin mutations, minimal attention has been paid to the potential diseases of cell–cell communication caused by mutations of Cx-associated molecules. An important Cx accessory protein is calmodulin (CaM), which is the major regulator of gap junction channel gating and a molecule relevant to gap junction formation. Recently, diseases caused by CaM mutations (calmodulinopathies) have been identified, but thus far calmodulinopathy studies have not considered the potential effect of CaM mutations on gap junction function. The major goal of this review is to raise awareness on the likely role of CaM mutations in defects of gap junction mediated cell communication. Our studies have demonstrated that certain CaM mutants affect gap junction channel gating or expression, so it would not be surprising to learn that CaM mutations known to cause diseases also affect cell communication mediated by gap junction channels.

## 1. Introduction

In most tissues, neighboring cells share charged and neutral molecules of low molecular weight via cell–cell channels clustered at gap junction membranes. This form of direct cell–cell communication enables tissues to coordinate many cellular activities [1,2]. Indeed, many diseases are known to result from abnormal gap junction mediated cell communication (see below).

A gap junction channel is formed by the interaction of two hemichannels (connexons/innexons) that form a hydrophilic pathway that crosses the plasma membranes of adjacent cells and a narrow extracellular space. Connexons/innexons are oligomers of six connexin/innexin proteins.

Gap junction channels are regulated by a chemical gating mechanism sensitive to cytosolic calcium concentration [Ca^2+^]_i_ and low pH_i_ [1,3,4,5,6,7] A large body of evidence has demonstrated that [Ca^2+^]_i_ in the range of 100 to low µM affects channel gating [1,8,9], suggesting that it is a fine modulator of direct cell–cell communication. Evidence for the effectiveness of nanomolar [Ca^2+^]_i_ suggests a role for Ca^2+^-modulated proteins where calmodulin (CaM) is the most likely candidate. Indeed, numerous studies have reported the role of CaM in gap junction channel gating and expression [1,9,10,11].

Since the early discoveries of the amino acid sequences of connexins (Cx) [12,13,14], numerous studies have identified diseases caused by connexin mutations [1,2,15,16,17,18]. In contrast, the potential role of CaM mutations in cell–cell communication has not been addressed. This is not surprising since, until recently, no one expected that mutations of a molecule like CaM, which is fundamental to life in plants and animals, would enable cell survival. In contrast, much to our surprise, in the past few years several diseases caused by CaM mutations (calmodulinopathies) have been identified [19]. None of these calmodulinopathy studies, however, has considered the potential effect of CaM mutations on gap junction function. Therefore, this review is intended to raise awareness of the potential effect of CaM mutations (and CaM mutations yet to be discovered) on direct cell–cell communication.

## 2. Gap Junction Channelopathies

The molecular cloning of connexins cDNA, first reported for the liver connexin (Cx32) [12,13] and soon after for the cardiac connexin Cx43 [14], allowed the gap junction field to discover diseases caused by Cx mutations. The report that the X-linked Charcot-Marie-Toot disease (CMTX1), a peripheral demyelinating disease, is caused by Cx32 mutations [20] opened the way to a wealth of discoveries that succeeded in identifying numerous genetic diseases caused by mutations in many different connexins (see Table 1). The diseases are either “non-syndromic” or “syndromic” (i.e., involving a multiple set of physical signs and symptoms.) They involve structural and functional abnormalities in nerves, the central nervous system, heart, skin, eyes, teeth, ears, bone, hair, nails and lymphatic system [2,15,16,17,18,21,22,23]. The following is a brief review of the major gap junction channelopathies, without laying claim to completeness.

### 2.1. Non-Syndromic Diseases

#### 2.1.1. X-Linked Charcot-Marie-Tooth (CMTX1) Disease––Cx32 Mutations

Cx32 (GJB1) is expressed mainly in myelinated axons at the paranodal regions of Ranvier nodes, where it forms reflexive gap junctions at the folds of Schwann cell membranes, allowing the exchange of ions and small metabolites among myelin lamellae. CMTX1 is caused by a mutation in the Cx32 gene [20]. At present, more than four hundred and fifty Cx32 mutations have been discovered [24]. They affect Cx32 expression, single-channel conductance and channel gating. Certain mutations in the first half of the Cytoplasmic Loop (CL1), or COOH-Terminus (CT) deletions, increase transjunctional voltage (Vj) and chemical gating sensitivity. Mutations in the second transmembrane (TM2) and CT domains increase hemichannel permeability, resulting in loss of cytosolic molecules and Ca^2+^ influx. CMTX1 patients suffer from distal muscle weakness, amyotrophy, reduced reflexes, *pes cavus* (high arch) and loss of peripheral sensitivity. These deficits, which first appear in young boys, are due to peripheral nerve demyelination. Since the gene is on the X-chromosome, this disease is more severe in hemizygous males than heterozygous females. 

#### 2.1.2. Pelizaeus–Merzbacher like (PMD; Leukodistrophy Hypomyelinating 2, Hereditary Spastic Paraplegia 44 (HSP) Autosomal Recessive, Hereditary Lymphedema type 1C (LMPH1C)––Cx47 Mutations

Cx47 (GJC1), earlier known as Cx46.6 (GJA12), is expressed in oligodendrocytes and couples them to astrocytes, creating a glial syncytium-like intercellular communication. Curiously, Cx47 does not interact with neighboring oligodendrocytes.

A number of Cx47 mutations cause Pelizaeus–Merzbacher-like disease, also known as Leukodistrophy Hypomyelinating 2 disease [25]. Some mutations prevent Cx47 insertion into the plasma membrane, so these Cx47 mutants remain in the endoplasmic reticulum (ER). A PMD variation is Spastic Paraplegia Autosomal. Patients suffering from PMD display nystagmus, delayed development, spasms and dysarthria. Magnetic Resonance Imaging (MRI) demonstrates intense white-matter signals resulting from replacement of myelin with a watery substance.

The Hereditary Spastic Paraplegia 44 (HSP) Autosomal Recessive [26] results in spasms and weakness of arms and legs, and urinary discomfort caused by hypertonicity of the bladder. Additional symptoms such as peripheral neuropathy, muscle wasting (amyotrophy), ataxia, seizures and dementia may also occur.

Hereditary Lymphedema type 1C (LMPH1C) [27] is caused by autosomal dominant mutations that damage the lymphatic system. The major consequence of this disease is chronic leg swelling, but occasionally swelling also affects the arms, face, chest and genitals. Often, lymphedema is associated with skin disorders (nail dysplasia or papillomatosis).

#### 2.1.3. Deafness Autosomal Recessive (1A) and Dominant (3A)–Cx26 Mutations, and Autosomal Dominant Deafness-2B (DFNA2B)–Cx31 Mutations

Cx26 is expressed in the epithelium and connective tissue of the organ of Corti in the cochlea, where it regulates the removal of potassium ions and the intracellular pH. Indeed, the release of ATP from connexin hemichannels in cochlear non-sensory cells has been proposed to be the main trigger for action potential activity in immature sensory inner hair cells (IHCs), which is crucial for the refinement of the developing auditory circuitry [28]. Among non-syndromic deafness diseases, the Autosomal Recessive Deafness (1A) [29,30] is the most frequent, while ~20% result from the Autosomal Dominant type (3A). Cx26 truncation or missense mutations are the cause of deafness. Most mutations result in complete loss of hearing, while the N206S mutation results in only moderate hearing loss. The V84L mutation results in total deafness as it inhibits channel permeability to inositol 1,4,5-trisphosphate (IP_3_) and blocks Ca^2+^ waves. Autosomal Dominant Deafness (3A) is also caused by mutations in the transmembrane (TM1) domain or the extracellular loop (EL1) [29,30].

Autosomal Dominant Deafness-2B (DFNA2B) [31], resulting in the progressive loss of high-frequency hearing, is more severe in males and caused by missense, nonsense and deletion mutations in the GJB3 (Cx31).

#### 2.1.4. Porokeratotic Eccrine Ostial and Dermal Duct Nevus (PEODDN)–Cx26 Mutations

PEODDN results from Cx26 mutations [32]. This rare disease is manifested by the development of linear papules and plaques in the palms and soles and is already present at birth. In some cases, papules and plaques also occur in the trunk, face and proximal extremities.

#### 2.1.5. Hydrotic Ectodermal Dysplasia 2 (HED2), Clouston Type–Cx30 Mutations

HED2 disease, also named Clouston Syndrome [33], is caused by Cx30 mutations and mainly affects nails, hair and skin. The nails are affected by thickening and dystrophy; hair is brittle, short and wiry and in time alopecia develops. Palmoplantar keratoderma may also occur, as well as clubbing fingers and joint hyperpigmentation.

#### 2.1.6. Erythrokeratodermia Variabilis Progressiva (EKVP)–Cx31, Cx30.3 and Cx43 Mutations

EKVP results in hyperkeratosis in plaques and areas of erythema. This autosomal-dominant type results from Cx31, Cx30.3 or Cx43 mutations [34,35,36,37]. Palmoplantar keratoderma also occurs in ~50% of cases.

#### 2.1.7. Atrial Familial Fibrillation (ATFB11) and Atrial Standstill Digenic (ATRST1)–Mutations of Cx40

Cx40 is expressed primarily in the cardiac atrium [38], while Cx43 and Cx45 (in trace amounts), are expressed mostly in the ventricles [39]. ATFB11 results in cardiac arrhythmia caused by abnormal firing at regions different from the sinus node (ectopic activity). Some arrhythmias result from lack of Cx40 expression [40] or missense mutations of Cx40 [41].

Some Cx40 mutations, also associated with α-subunit 5 mutations in the Na^+^ voltage-gated channel gene (SCN5A), result in familial Atrial Standstill Digenic (ATRST1) disease [41]. ATRST1 causes constant or sporadic lack of atrial contraction, resulting in fainting.

#### 2.1.8. Cataract (CTRCT14 and CTRCT1)–Mutations of Cx46 or Cx50, Respectively

Autosomal dominant cataracts (CTRCT14) caused by Cx46 mutations manifests itself in various forms, which include the following types of lens cataract: posterior polar, zonular pulverulent, nuclear coralliform, embryonal nuclear and Coppock-like cataracts [42,43,44]. Abnormalities in the lens, already detected in young children, appear as nuclear opacities bordered by cortical dust-like precipitates.

Several autosomal dominant cataracts (CTRCT1) are caused by Cx50 mutations [45,46]. The various types include: zonular pulverulent, nuclear progressive, nuclear pulverulent, stellate nuclear, nuclear total, total and posterior subcapsular. Areas of lens opacity are usually observed at birth and worsen during childhood. Microcornea [47] may also occur.

#### 2.1.9. Vascular Malformation. GJA4 (Cx37) Mutations

Mutations in Cx37 have recently been found to cause cutaneous and hepatic vascular lesions displaying enlarged vascular spaces, fibrous stromal formations, altered smooth muscle cells, hepatic hemangiomas (HHs) and malformations in skin veins [48]. The mutation G41C in the first transmembrane domain causes changes in cell morphology and activates the serum/glucocorticoid-regulated kinase 1 (SGK1) in a non-conventional way. Another mutation, G121T causes hepatic and cutaneous venous malformations.

### 2.2. Syndromic Diseases Caused by Connexin Mutations

#### 2.2.1. Deafness and Skin Disorders–Mutations of Cx26, Cx31, Cx30.3 and Cx30

All of these connexins are expressed in the cochlea. Their mutations result in both deafness and skin abnormalities.

##### Cx26 Mutations

There are two categories of syndromic deafness caused by Cx26 mutations. One includes Bart–Pumphrey Syndrome (BPS), Vohwinkel Syndrome (VS) and Keratoderma Palmoplantar with deafness Syndrome. The other includes Hystrix-like Ichthyosis with deafness Syndrome (HID) and Keratitis–Ichthyosis–deafness Syndrome (PPK).

BPS [49] results in deafness and leukonychia (white nail discoloration), with nail thickening and cracking. Finger and toe knuckles manifest wart-type growths and the skin of palms and soles becomes thicker (palmoplantar keratoderma).

VS [50] causes deafness and palmoplantar keratosis, characterized by increased skin thickness at palms and feet, with formation of honeycomb-like calluses. In addition, thick starfish-shaped patches develop in fingers, toes and knees. Eventually, fibrous tissue develops at fingers and toes, which limits blood flow so much that often spontaneous amputations occur. 

PPK [51] causes deafness and skin thickening in the palms and feet. In oocytes co-expressing Cx26 mutants and Cx43 wild-type, the Cx26 mutants also reduce Cx43 channel conductance, suggesting a direct interaction between Cx26 mutants and Cx43 [52].

HID [53,54,55], in addition to deafness, causes dry and scaly skin (ichthyosis), with the appearance of “porcupine quills” (hystrix), and hair loss (alopecia). HID also causes corneal inflammation (keratitis), which results in photophobia and eventual loss of vision [53,56]. These patients also develop palmoplantar keratoderma, reddened skin (erythrokeratodermia) and dry scaling skin (ichthyosis). Rarely, squamous-cell carcinomas may develop [57,58]. Only one Cx26 mutation causes HID: Cx26-D50N [54]; this mutation is also present in the KID syndrome, where it is the most common Cx26 mutation [59].

##### Cx31 and Cx30.3 Mutations

Mutations in Cx31 and Cx30.3, in addition to deafness, cause Erythrokeratodermia Variabilis et Progressiva (EKV) [34,36]. In EKV, the Cx31 mutations G2D, R42P and C86S prevent gap junction formation as they interfere with connexin trafficking to the plasma membrane, resulting in Cx31 accumulation into the ER [60].

##### Cx30 Mutations

Cx30 mutations can cause either Autosomal Dominant Deafness (3B) [61] or Autosomal Recessive Deafness (1B) [62], often associated with Ectodermal Dysplasia 2 (Clouston Syndrome) [33]. Combined Cx26 and Cx30 (GJB2/GJB6) mutations [63], or Cx26 and Cx31 (GJB2/GJB3) mutations [64], cause Digenic Deafness.

#### 2.2.2. Oculodentodigital Dysplasia (ODDD), Craniometaphyseal Dysplasia Autosomal Recessive (CMDR), Pantopalmar Keratoderma and Alopecia (PKA), Erythrokeratodermia Variabilis et Progressiva (EKVP), Syndactyly Type III––Cx43 Mutations

A number of the following disorders are caused by ODDD [65]: finger skin webbing (Syndactyly Type III), microphthalmia, craniofacial and dental alterations, microcephaly, alopecia, toes syndactyly, brittle nails, limb dysmorphisms, cleft palate, spastic paraplegia and neurodegeneration. In some cases, Cx43 missense mutations and a codon duplication affect connexon assembly and alter channel conduction. Syndactyly Type III can manifest itself either individually or in conjunction with ODDD. Recent evidence for ODDD mutations at the Cx43 CaM binding site in the CL domain [66] suggests a possible role of CaM in some cases of ODDD (see below).

The autosomal recessive disease CMDR [67] causes hyperostotic craniofacial abnormalities such as protrusion of lower jaw, midface hypoplasia, wide nasal bridge, prominent forehead, increased eye spacing, and wide and long bones with metaphyseal flaring.

PKA [68] results in skin thickening in the palms and soles, toenail abnormalities, skin discoloration, and alopecia. PKA may also be caused by mutations of Cx26 and Cx30.

EKVP [37] results in hyperkeratotic plaques and areas of erythema. Mutations in Cx31 and Cx30.3 (see in the previous) may also cause this autosomal-dominant disease, but patients with Cx43 mutations also manifest enlarged porcelain-white lunulae (white half-moon at the cuticle) and peri-orificial darkening.

### 2.3. Does Calmodulin Play a Role in Gap Junction Channelopathies?

While connexin mutations causing diseases may involve CaM-binding domains, thus far little interest has been devoted to the potential role of CaM mutants in gap junction channelopathies. In one case, however, there is evidence that mutations at the CL2 CaM-binding site of Cx43 play a role.

In patients with Oculodentodigital Dysplasia (ODDD), over one-third of the 73 known mutations are localized in the CL2 domain, at or near the most relevant CaM binding site. This domain comprehends the following sequence:

Res. 131–EIKKFKY***GIEEHGKVKMRGGLLRTYII***SILFKSIFE–166

of which the sequence E141-I157 (in bold and italic characters) is most relevant for CaM binding. Residues mutated in ODDD patients include: G138D/R/S, G143S/D, K144E, V145G, M147T, R148Q/G, and T154A/N [66,69,70,71,72] (see in the previous, in red letters). The mutations G138R, G143S, and T154A have been reported to affect hemichannel function and inhibit gap junction communication [66,69,70,71,72]. Curiously, mimetic peptides containing these mutations still bind CaM [66]. Therefore, either the CaM interaction to the Cx43 mutant is abnormal or the mutation affects channel formation via a CaM-independent mechanism. However, in the analysis of the CaM binding site prediction, the CaM binding site of the G138R, G143S and T154A mutant, identified by a computer program that rate the sites 0-9 [73], is shifted in the NH_2_ direction by a few residues (see in the following). In contrast, in most of the other ODDD mutants tested the CaM binding site is shifted in the COOH direction. Whether these shifts are functionally relevant is hard to predict. Below are the binding predictions of the G138R, G143S, T154A mutant found to cause ODDD:

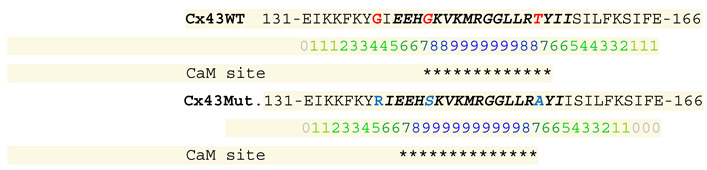



Significantly, the absence of gap junction communication in cells expressing the G138R–G143S–T154A mutant is due to fact that this mutant resulted in increased intracellular Cx43 localization and that the Cx43 mutant present in the plasma membrane did not form gap junctions [66], which is consistent with evidence that cells expressing this ODDD mutant are unable to exchange Lucifer Yellow [66]. The inability of the G138R–G143S–T154A mutant to form gap junction is consistent with evidence for the role of CaM in gap junction formation [1,74] (see below). Also consistent with the relevance of the CL2 CaM-binding site is evidence that the deletions of 5 to 6 residues within this domain prevents the formation of functional gap junction channels [70]. Furthermore, mammalian cells expressing the Cx43 CL2 mutant T154A showed the absence of dye coupling [71]. In a study [72], cultured fibroblasts expressing the Cx43 CL2 mutated at G138R and G143S showed slower growth, decreased migration and altered cell polarization, while the Cx43 variant in the plasma membrane, gap junction plaques, intercellular communication and hemichannel activity were altered.

## 3. Calmodulinopathies

Calmodulin (CaM) is the major mediator of cellular functions regulated by cytosolic calcium [75]. In addition to activating numerous enzymes, CaM is involved in the function of a growing family of membrane channels that, in addition to connexins/innexins channels, includes voltage-gated Ca^2+^ channels (Ca_V_1.2), Na^+^ channels (Nav1.5), and K^+^ channels K_V_7.1(KCNQ1) and K_V_11.1 (KCNH2); small conductance Ca^2+^-activated K^+^ channels (SK); inwardly rectifying K^+^ channels (Kir, IRK); cyclic nucleotide-gated channels (CNG); ryanodine receptor (RyR2); transient receptor potential channels (TRP) [76,77,78]; and the water channel aquaporin-0 (AQP0), also known as the eye lens protein MIP26 [79,80,81], the CaM binding site of which at the COOH-terminus (res. 223–241) we identified in the mid-1980s [82]. Recent evidence of diseases caused by CaM mutations [83] suggests the potential role of CaM mutants in diseases affecting gap junction function.

Even though CaM mutations were identified over two decades ago in Paramecium [84] and later in Drosophila [85], some of which are lethal [85], until a few years ago no one expected that CaM mutants would be expressed in humans and not be lethal. Indeed, eight years ago Mette Nyegaard and coworkers made the startling discovery that several members of a Swedish family suffering from severe cardiac dysfunctions such as syncope and cardiac arrest were affected by a CaM mutation [83]. This surprising discovery is well described in an excellent review article by Jensen and coworkers [19] as follows: “The critical role of accurate Ca^2+^ signaling on cellular function is underscored by the fact that there are three independent CaM genes (CALM1-3) in the human genome. All three genes are functional and encode the exact same CaM protein. Moreover, CaM has a completely conserved amino acid sequence across all vertebrates. Given this degree of conservation, it was long thought that mutations in CaM were incompatible with life. It was therefore a big surprise when the first CaM mutations in humans were identified six years ago. Today, more than a dozen human CaM missense mutations have been described, all found in patients with severe cardiac arrhythmias” [19]. For reviews on calmodulinopathies see [19,86,87].

**Figure 1:**
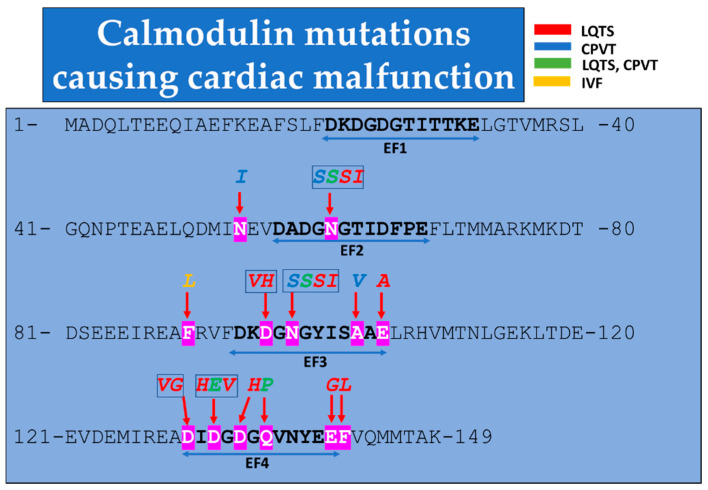
Calmodulin sequence depicting mutations known to cause cardiac diseases such as Long QT Syndrome (LQTS), Catecholaminergic Polymorphic Ventricular Tachycardia (CPVT) and Idiopathic Ventricular Fibrillation (IVF). The double-headed arrows indicate the Ca^2+^-binding sequences (EF1-EF4).

Almost two dozen CaM mutations have been found to cause cardiac malfunctions (Table 2 and Figure 1). Note that the CaM residues are labeled based on the Human Genome Variation Society’s nomenclature, HGNS [88]). Most of them occur in the C-lobe, one in the N-lobe (CaM–N54I) and one in the linker between C- and N-lobes (CaM–F90L). In most cases, the electrocardiogram (ECG) demonstrates the presence of Long QT Syndrome (LQTS), a change that affects the electrical activity of the heart, which is often associated with Catecholaminergic Polymorphic Ventricular Tachycardia (CPVT) phenotype and Idiopathic Ventricular Fibrillation (IVF). CPVT patients manifest ventricular tachycardia that can lead to death by ventricular fibrillation.

In most cases, cardiac malfunctions have been attributed to the effect of CaM mutations on the function of the ryanodine receptor (RyR2) and the cardiac L-type voltage gated Ca^2+^ channel Ca_V_1.2. But, other membrane channels, potential targets of CaM mutants, have also been suggested: the K^+^ channels K_V_7.1 (KCNQ1) and K_V_11.1 (KCNH2), the Na^+^ channel Na_V_1.5 (SCN5A), as well as the Ca^2+^/CaM-dependent kinase II (CaMKII) [19,86,87]. Curiously, however, in spite of three decades of evidence for a direct CaM role in gap junction channel regulation [1,9,89,90], the potential consequences of these CaM mutants on direct cell–cell communication––a mechanism fundamental for the function of virtually all vertebrate and invertebrate organs––have not yet been addressed [19,86,87].

The CaM mutants reported thus far include one N-lobe mutant, one mutant in the flexible linker and 16 mutants in the C-lobe, all of which are located in the Ca^2+^-binding sequences (Figure 1) [19,86]. 

## 4. Do calmodulin Mutants Affect Gap Junction Channel Function?

For the past four decades, CaM has been reported to play a role in gap junction function. In the early 1980s, Johnston and Ramón found that internally perfused crayfish lateral giant axons do not uncouple when [Ca^2+^]_i_ is increased or pH_i_ is lowered below normal [91]. Based on these findings, which were confirmed by Arellano and coworkers [92], they proposed that a soluble intermediate mediates the effect of Ca^2+^/H^+^ on gap junction channel gating. In 1981, we first reported in a Cell Biology Meeting’s abstract the CaM role in gap junction channel gating due to the inhibitory effect of a CaM blocker on the electrical uncoupling of *Xenopus* embryonic cells; the formal paper was published in 1983 [93]. The role of CaM on gating was also supported by evidence that CaM binds to Cx32 and to gap junction fragments from crayfish hepatopancreas [94,95]. The CaM role in gating has been confirmed over the years by data generated by various experimental methods such as: treatments with CaM blockers, inhibition of CaM expression, expression of CaM mutants, immunofluorescent co-localization of CaM and gap junctions, evidence for the presence of CaM binding sites in connexins and in vitro data for CaM interaction with connexins or connexin peptide CaM binding sites, among others [1,9,89,90].

Therefore, one may ask: do the CaM mutations recently found to cause cardiac diseases also affect direct cell–cell communication? While no one has yet tested the potential effect on the gap junction function of CaM mutants recently reported to cause cardiac malfunction such as LQTS, CPVT or IVS (see above), we know that most of the CaM mutants we tested indeed affect gap junction channel gating and expression [96,97,98]. The CaM mutants we tested on gap junction function are CaMCC, CaMNN, CaM1,2,3,4, CaM1,2 and CaM3,4 (see below) [1,96].

### 4.1. Expression of the CaM Mutants CaMCC or CaMNN

In CaMCC, the NH_2_-terminal EF-hand pair (res. 9–76) of CaM is replaced by the COOH-terminal pair (res. 82–148). The Ca^2+^ affinity of the COOH-terminal EF-hand pair is much greater than that of the NH_2_-terminal pair [99]; therefore, we thought that the expression of CaMCC might increase the gating sensitivity of gap junction channels to [Ca^2+^]_i_.

**Figure 2:**
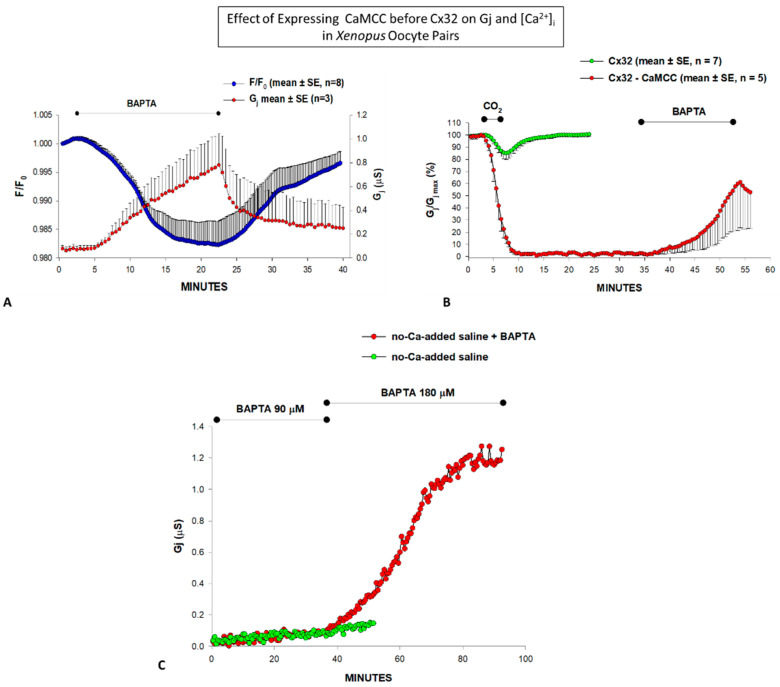
In oocytes expressing Cx32 after CaMCC, the junctional conductance (Gj) was very low (**A**), but drastically increased with BAPTA (180 µM) superfusion (**A**–**C**) as [Ca^2+^]_i_, dropped (A, F/F0). Lower [BAPTA] (90 µM) were minimally effective (**C**). The channels were more sensitive to 100% CO_2_ than controls ((**B**) and Figure 3), as Gj rapidly dropped to zero with CO_2_ applications as short as 3 min (**B**), while in controls, Gj decreased by only ~15% even with CO_2_ applications as long as 15 min (see Figure 3). After CO_2_ washout, Gj/Gjmax remained at 0% (**B**) but rapidly increased with 180 µM BAPTA superfusion (B) [100].

Indeed, in oocytes expressing CaMCC before Cx32, the junctional conductance (Gj) is minimal, but greatly and reversibly increases when [Ca^2+^]_i_ is lowered by BAPTA (180 µM) superfusion (Figure 2A) [97,100]. CaMCC enhances Ca^2+^ gating sensitivity to such an extent that gating is even activated by resting [Ca^2+^]_i_ (Figure 2C), most likely because the CaM C-lobe rather than the N-lobe is now the gating lobe. The enhanced gating sensitivity induced by CaMCC expression was further confirmed by testing the effect of 100% carbon dioxide (CO_2_), which caused a drop in pH_i_ to ~6.3 and increased [Ca^2+^]_i_ [100].^.^ In the presence of CO_2_, Gj rapidly dropped to zero, while in controls it decreased by only ~15% (Figure 2B). With CO_2_ washout, Gj remained at zero indefinitely, but began to recover, reversibly, with BAPTA (180 µM) superfusion (Figure 2B), which obviously decreased [Ca^2+^]_i_ below resting values (Figure 2B). Significantly, lower BAPTA concentrations (90 μM) are minimally effective (Figure 2C) [100].

**Figure 3:**
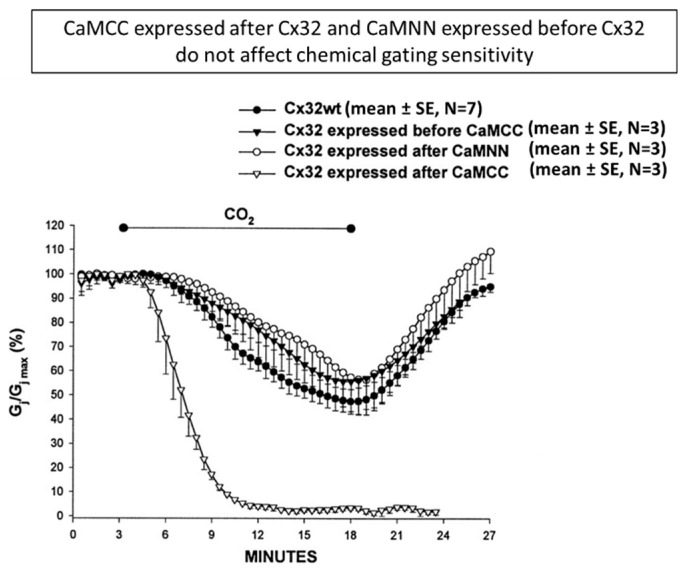
In oocytes expressing Cx32 after CaMCC, Gj rapidly dropped to zero with 15 min CO_2_ applications, while in controls it decreased by only ~50%. In contrast, expression of Cx32 before CaMCC or expression of CaMNN had no effect on gating [100].

The effect of CaMCC was only seen when CaMCC was expressed before Cx32 [100] (Figure 3), suggesting that CaMCC effectively competed with CaM wild-type for Cx32 binding. In contrast, CaMNN, in which the NH_2_-terminal pair (res. 9–76) of CaM replaced the COOH-terminal pair (res. 82–148), had no effect (Figure 3). This suggested that CaMNN was unable to compete against wild-type CaM88 for Cx32 binding. The sequence of CaMCC-Cx32 expression indicated that CaMCC bound to Cx32 before connexon assembly [100].

**Figure 4:**
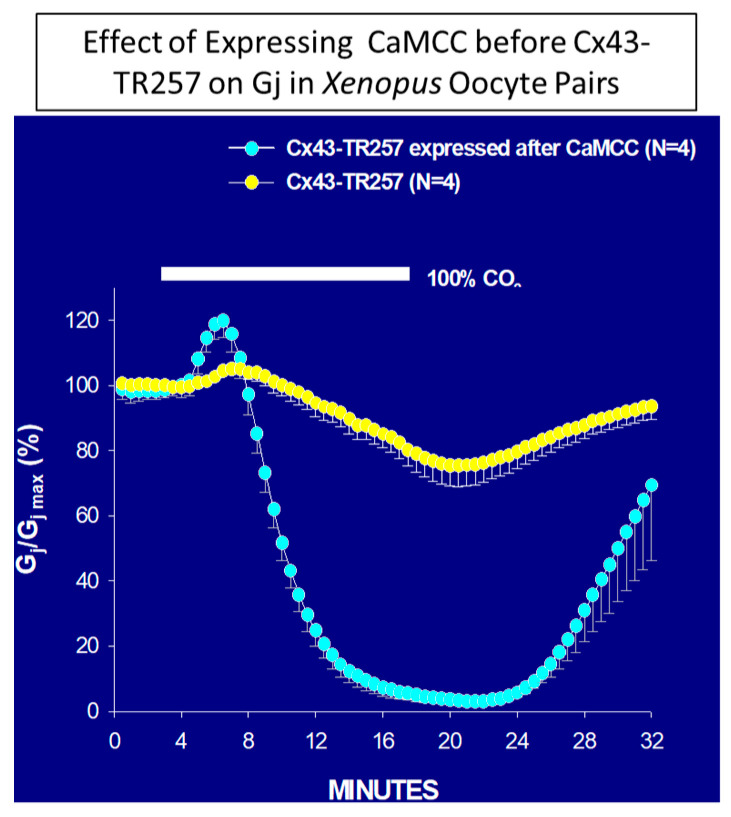
CT-deleted Cx43 channels (Cx43TR257), expressed after CaMCC, were much more sensitive to CO_2_ than controls, as Gj rapidly dropped to nearly 0% with 15 min of 100% CO_2_ application, while in controls, Gj decreases by only ~25% [90].

The same results were obtained in oocytes expressing Cx43 or COOH-terminus (CT) deleted Cx43 (Cx43-TR257) after CaMCC expression (Figure 4) [90]. As with Cx32 channels, in some experiments Cx43–TR257 channels expressed after CaMCC remained indefinitely closed following CO_2_ washout, but Gj reversibly recovered with BAPTA (180 µM) superfusion [90], as it did with Cx32 channels (Figure 2B). 

### 4.2. Expression of the CaM Mutants CaM1,2,3,4, CaM1,2 or CaM3,4

**Figure 5:**
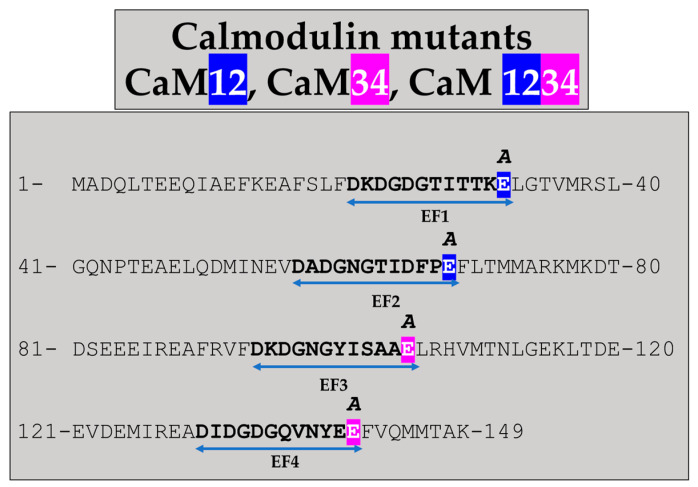
Calmodulin mutants expressed before Cx32, tested as potential inhibitors of gap junction expression. CaM1,2,3,4 (E32A, E68A, E105A, E141A), CaM1,2 (E32A, E68A) and CaM3,4 (E105A, E141A). The double-headed arrows indicate the sequence of the four Ca^2+^-binding sites (EF1–EF4).

CaM mutants lacking one or more of the four high affinity Ca^2+^-binding sites were expressed before Cx32 expression. In these CaM mutants, glutamates (E) relevant for Ca^2+^ binding were replaced by alanine (A) in the CaM EF-hand domains (Figure 5). In CaM there were four Ca^2+^-binding sites: #1, res. 21–32; #2, res. 57–68; #3, res. 94–105; #4, res. 130–141 (Figure 5). These E to A mutations were shown to dramatically reduce the Ca^2+^ affinity of the CaM EF-hand loops [101].

**Figure 6:**
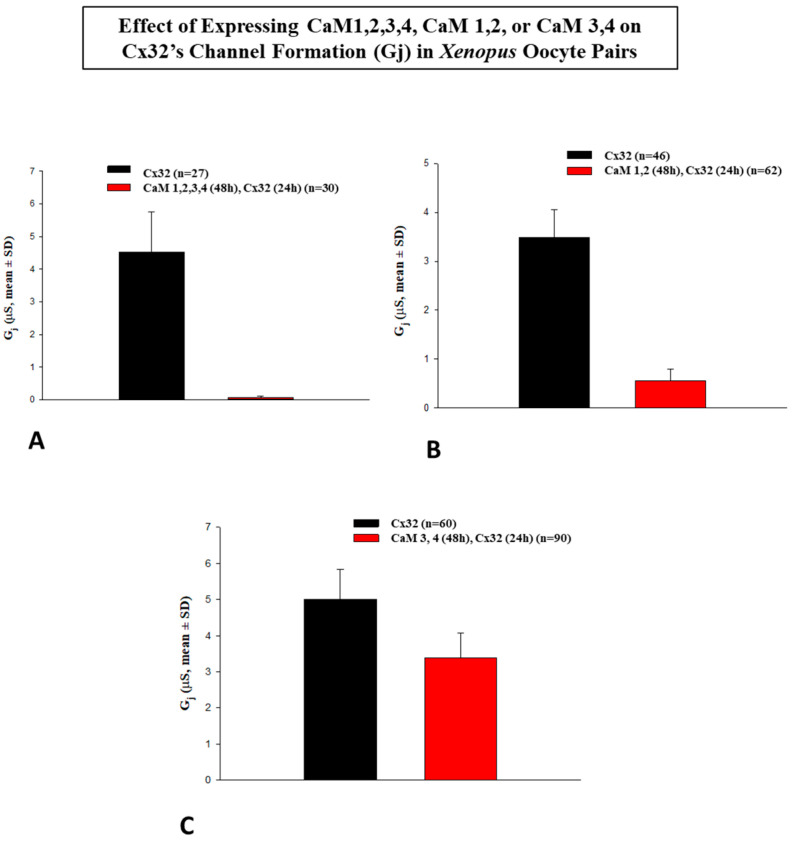
Junctional conductance (Gj) monitored in Xenopus oocyte pairs expressing Cx32 after the expression of CaM mutants lacking one or more of the four high-affinity Ca^2+^-binding sites (see Figure 5). The expression of CaM1,2,3,4 (E32A, E68A, E105A, E141A, (**A**)) or CaM1,2 (E32A, E68A, (**B**)) effectively inhibits Cx32-channel formation. In contrast, expression of CaM3,4 (E105A, E141A, (**C**)) is not significantly effective. This indicates that Ca^2+^-activation of the CaM N-lobe is relevant for gap junction formation.

Expression of CaM1,2,3,4 (E32A, E68A, E105A, E141A) or CaM1,2 (E32A, E68A), before expression of Cx32, effectively blocked the formation of functional Cx32 channels (Figure 6A,B) whereas expression of CaM3,4 (E105A, E141A) had no significant effect (Figure 6C) [1,96]. The effectiveness of CaM1,2,3,4 and CaM1,2 in inhibiting gap junction formation indicated that both of them successfully competed with wild-type CaM in binding to the CaM binding sites of Cx32. Since CaM3,4, unlike CaM1,2, allows almost normal expression of functional gap junctions, it seemed likely that Ca^2+^-activation of the CaM N-lobe was the most relevant for gap junction formation. Evidence that CaM1,2,3,4 effectively competed with wild-type CaM further supported the idea that CaM binds to connexins in a Ca^2+^-independent way [102]. Furthermore, the fact that CaM1,2, but not CaM3,4, prevented channel formation indicated that normal Ca^2+^-affinity of the EF hand domains of the CaM N-lobe was needed for gap junction formation. Alternatively, although unlikely, it is possible that the effect of CaM1,2 and CaM1,2,3,4 resulted from the prevention of gap junction channel opening.

As a hypothesis, we proposed that the C-lobe of CaM binds to a Cx32 site at the resting [Ca^2+^]_i_ (~50 nM), or even in Ca^2+^-independent way [102]. With a [Ca^2+^]_i_ rise above ~50 nM, Ca^2+^ binds to the N-lobe enabling it to interact with the same or another Cx32 CaM-binding site, initiating Cx oligomerization in hexameric connexons. Indeed, the interaction of CaM and connexins has been found to be essential for connexin oligomerization into connexons [74]; this study, which used an in vitro, cell-free synthesis system, demonstrated that the formation of Cx32 hexameric connexons is reversibly inhibited by a calmodulin-binding synthetic peptide and the CaM inhibitor W7 [74].

CaM3,4, a mutant that lacks Ca^2+^-binding sites at the C-lobe, does not significantly inhibit the formation of gap junction channels (Figure 6C). Therefore, it seems likely that the majority of CaM mutants that cause cardiac diseases, which affect the C-lobe’s Ca^2+^-binding site (see in the previous), may not significantly interfere with cardiac gap junction formation, although they may affect channel gating.

The CALM1 N54I mutant (Table 2, and Figure 1), which causes Catecholaminergic Polymorphic Ventricular Tachycardia (CPVT), may interfere with gap junction formation because this residue is in the CaM N-lobe and just precedes the second CaM Ca^2+^-binding site (EF2, Figure 1; see in the previous). However, since N54 is located in the solvent exposed surface of the N-lobe in alpha-helix 3, its mutation may not affect the second N-lobe Ca^2+^-binding site [78].

## 5. Conclusions and Future Perspective

Thus far, most of the research on diseases caused by defects of direct cell–cell communication via gap junction channels has focused on connexin mutants. However, evidence that connexins interact with several cytoplasmic components [103] that may be subjected to mutation has been thus far mostly ignored. One of the goals of this review article was to raise awareness of potential diseases caused by the accessory components of connexins. While thus far there is no evidence that cardiac diseases caused by CaM mutants that alter cardiac functions (Table 1) also involve gap junction channel function, future studies should consider the potential role of these and other CaM mutations in gap junction regulation or expression. It is somewhat surprising that the CaM mutants causing cardiac diseases did not affect cell–cell communication, an essential element of cardiac function. In view of the importance of CaM in cell function, it is also surprising that CaM mutations, which alter so dramatically cardiac function, have not been reported to alter the function of cells other than cardiac cells. It is hard to predict the effect, if any, of the pathological CaM mutations on gap junction expression or gating permeability. However, the reported pathological mutations in the CaM C- or N-lobe are likely to affect both connexin expression and chemical gating.

For testing the potential effect of CaM mutants on gap junction channels, one may want to study the in vitro binding of the mutants to peptides matching the CaM binding sites identified in the connexin NT, CL2 and CT domains. In addition, their effect on gap junction channel gating or expression should be tested by expressing them in oocyte pairs or small cultured cells expressing different connexins.

## Figures and Tables

**Table 1:** Genetic Diseases Caused by Connexin Mutations.

GENE	PROTEIN	ORGAN/SYSTEM	DISEASE
*GJA1*	Cx43 (syndromic)	Bone	Craniomethaphyseal Dysplasia (autosomal recessive)
		Skin	Erythrokeratodermia Variabilis et Progressiva (EKVP),
		Eye	Palmoplantar Keratoderma and Alopecia 1 (PPKCA1),
		Hair	Oculodentodigital Dysplasia (autosomal recessive)
			Syndactyly type III
*GJA3*	Cx46 (nonsyndromic)	Eye	Cataract
*GJA4*	Cx37 (nonsyndromic)	vessels	Vascular malformation
*GJA5*	Cx40 (nonsyndromic)	Heart	Atrial Familial Fibrillation 11 (ATFB11),
			Atrial Standstill Digenic (GjA5/SCN5A)
*GJA8*	Cx50 (nonsyndromic)	Eye	Cataract
*GJB1*	Cx32 (nonsyndromic)	Nervous System	Charcot-Marie-Tooth Neuropathy, X-Linked 1
*GJB2*	Cx26 (syndromic)	Skin + Ear	Keratitis-Ichthyosis (with deafness) Syndrome,
			Vohwinkel Syndrome,
			Palmoplantar Keratoderma (PKK) (with deafness),
			Hystrix-like Ichthiosis (with deafness),
			Bart-Pumfrey syndrome (with knuckle pads, leukonykia and deafness)
	Cx26 (nonsyndromic)	Skin or Ear	Portokeratotic Eccrine Ostial and Dermal Duct Nevus (PEODDN)
			Deafness, Autosomal Dominant (3A),
			Deafness, Autosomal Recessive (1A),
			Deafness Digenic (GjB2/GjB6)
*GJB3*	Cx31 (nonsyndromic)	Ear	Deafness Digenic (GjB2/GjB3),
			Autosomal Dominant Deafness-2B (DFNA2B)
	Cx31 (syndromic)	Ear + Skin	Erythrokeratodermia Variabilis et Progressiva (EKVP), with deafness
*GJB4*	Cx30.3 (syndromic)	Ear + Skin	Erythrokeratodermia Variabilis et Progressiva (EKVP) with deafness
*GJB6*	Cx30 (syndromic)	Ear + Skin	Autosomal Dominant Deafness (3B),
			Autosomal Recessive Deafness (1B)
			Digenic Deafness GjB2/GjB6)
	Cx30 (nonsyndromic)	Skin	Hydrotic Ectodermal Dysplasia 2 (HED2),
			Clouston Type
*GJC2/* *(GJA12)*	Cx47 (nonsyndromic) (Cx46.6)	Nervous System or Lymphatic System	Pelizaeus-Merzbacher-like (Leukodystrophy Hypomyelinating 2), Hereditary Spastic Paraplegia 44, Autosomal Recessive (HSP), Hereditary Lymphedema 1C

**Table 2:** Calmodulin mutants causing cardiac diseases (HGVS nomenclature).

CaM Mutation	Mutated Gene	Diagnosis
N54I	CALM1	CPVT
F90L	CALM1	IVF
D96H	CALM2	LQTS
D96V	CALM2	LQTS
N98I	CALM2	LQTS
N98S	CALM1, CALM2	LQTS ± CPVT
A103V	CALM3	CPVT
E105A	CALM1	LQTS ± CPVT
D130G	CALM1, CALM2, CALM3	LQTS
D130V	CALM2	LQTS
D132E	CALM2	CPVT + LQTS
D132H	CALM2	LQTS
D132V	CALM1	LQTS
D134H	CALM2	LQTS
Q136P	CALM2	CPVT + LQTS
E141G	CALM1	LQTS
E141K	CALM3	LQTS
E141V	CALM1	LQTS
F142L	CALM1, CALM3	LQTS
